# Hardness-Dependent Freshwater Quality Criteria for the Protection of Aquatic Organisms for Cadmium in China

**DOI:** 10.3390/toxics12120892

**Published:** 2024-12-08

**Authors:** Zeya Zhang, Rui Huang, Zhongjie Shen, Yili Fan, Chenglian Feng, Yingchen Bai

**Affiliations:** 1 State Key Laboratory of Environmental Criteria and Risk Assessment, Chinese Research Academy of Environmental Sciences, Beijing 100012, China; z.craes@163.com (Z.Z.); huangrui233@mails.ucas.ac.cn (R.H.); 17851550286@163.com (Z.S.); fanyl@craes.org.cn (Y.F.); fengcl@craes.org.cn (C.F.); 2 School of Environmental Science and Engineering, Changzhou University, Changzhou 213164, China; 3 College of Water Science, Beijing Normal University, Beijing 100875, China

**Keywords:** cadmium, freshwater quality criteria, water hardness, species sensitivity distribution

## Abstract

Cadmium poses a significant threat to freshwater aquatic organisms and ecosystems, making it essential to establish regional freshwater quality criteria (FWQC) in China to safeguard these organisms. The toxicity database for cadmium covered 249 acute toxicity data from 52 species (seven phyla and 27 families) and 62 chronic toxicity data from 21 species (four phyla and 12 families). During short-term exposure, *Morone saxatilis* displayed the most sensitivity to cadmium, whereas *Daphnia magna* showed the most sensitivity in long-term exposure scenarios. Significant correlations were identified between water hardness and the toxicity data for cadmium, with the acute toxicity coefficient (K_ATD_) at 1.0227 (n = 52, *p* < 0.05) and the chronic toxicity coefficient (K_CTD_) at 0.4983 (n = 21, *p* < 0.05). With the species sensitivity distribution method, the short-term freshwater quality criteria (S-FWQC) were derived with a normal distribution as the best fit (*R*^2^ 0.9793), while the long-term freshwater quality criteria (L-FWQC) were calculated using a logistic distribution as the best fit (*R*^2^ 0.9686). The formulas for the S-FWQC and L-FWQC were represented as $10^{\left(1.0227\times lg(H)-1.5444\right)}$ and $10^{\left(0.4983\times lg(H)-1.7549\right)}$, respectively, with water hardness serving as an independent variable. This study offers valuable insights for improving the management of cadmium to protect freshwater aquatic organisms in China.

## 1. Introduction

Due to its high energy density, corrosion resistance, and optoelectronic properties, cadmium, as a non-essential element and common pollutant of heavy metal, was widely utilized in industries such as batteries, pigments, solar cells, etc. [1,2,3]. With large-scale industrial applications, cadmium inevitably entered natural water systems during production, usage, and disposal [4,5,6]. Previous studies showed that cadmium was a prevalent environmental contaminant with both acute and chronic toxicity to aquatic organisms [7,8]. For example, long-term exposure to low environmental doses of cadmium of 0.1–1 μg/L was demonstrated to cause damage to the gills, muscles, brain, and intestines of *Oreochromis mossambicus* at 28–32 °C and dissolved O_2_ of 5 mg/L [9]. Concurrently, cadmium can affect metal ion binding, oxidative stress, and energy metabolism within *Pectinidae* tissues in seawater equipped with an air pump [10]. Histopathological changes in the liver and kidney tissues of *Morone saxatilis* were induced by following 96 h exposure to cadmium at a concentration of 20 µg/L [11]. Furthermore, in the 30 d chronic toxicity tests of cadmium (7.5 µg/L) on *Daphnia magna*, tissue damage was induced, leading to stunted growth and reduced reproductive capacity in *Daphnia magna* [12]. Due to its hazardous properties, cadmium was classified as a priority pollutant to freshwater aquatic organisms and human health in several countries including the USA, Canada, Australia, New Zealand, etc., as well as international organizations including the European Union (EU) [13,14,15,16]. The freshwater quality criteria (FWQC) for the protection of aquatic organisms were defined as the maximum allowable concentrations of pollutants in water to ensure the health and safety of aquatic organisms [17,18,19]. As the scientific foundation of water quality standards (WQSs), the FWQC play an important role in environmental management. The FWQC for the protection of aquatic organisms for cadmium were investigated and established in several countries, including the USA, Canada, Australia, and New Zealand [20,21,22].

WQSs for freshwater in China were formulated by adopting and referring to the FWQC of developed countries/international organizations [17,23,24]. For instance, 0.001 mg/L for Class I, 0.005 mg/L for Class II-IV, and 0.01 mg/L for Class V were set in environmental quality standard for surface water (GB3838-2002) [25] based on the criteria and WQSs of the USA (FWQC for aquatic life of 0.001 mg/L, human health FWQC of 0.005 mg/L, WQS for drinking water sources of 0.01 mg/L, and agricultural irrigation WQS of 0.005 mg/L), Japan (drinking WQS of 0.01 mg/L and WQS for drinking water sources of 0.01 mg/L), the UK (drinking WQS of 0.01 mg/L), etc. [26]. However, the applicability and scientific accuracy of the FWQC have been questioned in protecting the bio-environmental system because of the significant differences among other countries and the geographical and climatic conditions, aquatic biota, etc. in China [18,23,27,28]. For instance, the families of *Salmonidae* and *Cyprinidae* were proposed for the FWQC for the protection of aquatic organisms in the USA and China, respectively, because of the variations in freshwater biota among different nations [29,30]. The FWQC for the protection of aquatic organisms for cadmium, ammonia, and phenol were issued by the Ministry of Ecology and Environment (MEE) in China in 2020 [31,32,33]. With the processes of research, many research studies indicated that invasive species and internationally common species without distribution in China should be ruled out when deriving FWQC. Therefore, the FWQC for the protection of aquatic organisms from cadmium should be revisited based on the current research results and current guidelines to ensure the scientific and effective management of cadmium in freshwater environments in China [30].

The species sensitivity distribution (SSD) method was successfully applied to derive the FWQC of various pollutants in several countries, including the USA and Canada [20,21]. The SSD method assumed that the tested species were collected randomly and representative of all aquatic organisms within the freshwater ecosystem. The SSD method had clear advantages due to its support by statistical theory and its ability to utilize a full array of toxicological data of freshwater aquatic organisms [34,35]. Therefore, the SSD method might serve as a valuable tool for deriving the FWQC for the protection of aquatic organisms for cadmium in China [36,37,38,39]. In addition, the toxicity of cadmium to aquatic organisms was affected by water quality parameters, such as hardness, temperature, pH, etc. [32,40,41]. As water hardness increased, the toxicity of cadmium decreased in aquatic organisms, which was attributed to competition between heavy metals and Ca^2+^ and Mg^2+^ ions for binding sites on the cell membrane [29,42,43]. The median effect concentration (EC_50_) of cadmium to *Morone saxatilis* in soft water (3.7 μg/L, 40 mg/L as CaCO_3_) was 86.3% less than that in hard water (27.0 μg/L, 285 mg/L as CaCO_3_), demonstrated by 96 h acute toxicity tests [44]. For instance, previous studies indicated that higher calcium concentrations can reduce the acute toxicity and uptake of cadmium in *Daphnia magna* with calcium concentrations of 0.5–200 mg/L at pH 8.00–8.20 [45]. Significantly positive relationships have also been documented between the water hardness of experimental water and the toxicity of cadmium to aquatic organisms over the USA by the USEPA [29]. Therefore, it is necessary to derive the hardness-dependent FWQC for the protection of aquatic organisms for cadmium in China.

The objectives of this research included the following: (1) to compare the sensitivities of various species by establishing a toxicity database for cadmium to aquatic organisms, (2) to determine the quantitative relationship between the toxicity data (TD) of cadmium to aquatic organisms and water hardness, and (3) to derive the FWQC for the protection of aquatic organisms for cadmium in China.

## 2. Materials and Methods

### 2.1. Toxicity Data Collection and Screen

With the development of environmental criteria research and the increasing amount of acute toxicity data (ATD) and chronic toxicity data (CTD) for pollutants, stricter requirements on the selection of aquatic organisms were imposed by the technical guidelines for deriving water quality criteria for freshwater organisms. The guideline highlighted that tested species should reflect the characteristics of freshwater biota in China, and invasive species such as *Oreochromis niloticus*, *Gambusia affinis*, *Procambarus clarkia,* and others should not be considered as test species. To derive the FWQC for cadmium, aquatic species in China were screened based on documents such as the Chinese biodiversity catalog, the recommended species list for deriving freshwater quality criteria in China, and high-quality scientific papers published on China’s national knowledge infrastructure and science citation index. During the derivation of FWQC, the short-term freshwater quality criteria (S-FWQC) and long-term freshwater quality criteria (L-FWQC) were established to provide an appropriate level of protection for aquatic organisms with ATD and CTD, respectively. Short-term exposure was associated with acute toxicity, typically characterized by exposure durations spanning from 24 to 96 h. In the derivation of FWQC for cadmium, the preferred times of short-term exposure are 24, 48, and 96 h for Rotifera, Daphnia/midges, and other organisms, respectively [31]. The median lethal concentration (LC_50_) and EC_50_ were chosen as endpoints for short-term exposure experiments for cadmium to freshwater aquatic organisms [18]. Long-term exposure, on the other hand, refers to chronic toxicity, with exposure durations designed to be at least 21 days or spanning across one or more generations. The no observed effect concentration (NOEC), the lowest observed effect concentration (LOEC), the maximum acceptable toxicant concentration (MATC), the 10% effect concentration (EC_10_), and the 20% effect concentration (EC_20_) were typically chosen as endpoints for the long-term exposure of chronic toxicity during FWQC derivation [34]. Due to their rapid cell division rates, an exposure time of four days was selected as chronic toxicity for algae, including *Chlorella vulgaris*, *Scenedesmus acutus*, etc. [18]. When the LOEC and NOEC were obtained under the same experimental conditions, the MATC was calculated as the geometric mean of the NOEC and LOEC to derive the FWQC for cadmium in our investigation. A list of abbreviations can be found in the Supplementary Materials (Table S1).

During the derivation of FWQC, both ATD and CTD of cadmium to aquatic organisms were collected from the literature and toxicity databases. In detail, the retrieval strategy of “TI = (Cd ion or Cadmium) AND TS = (toxicity or LC_50_ or EC_50_ or EC_20_ or EC_10_ or NOEC or LOEC or MATC)” was applied to the Chinese knowledge resource integrated database (http://www.cnki.net/, accessed on 4 August 2024), Elsevier (http://www.sciencedirect.com, accessed on 13 August 2024), and Web of Science (http://www.webofscience.com, accessed on 15 August 2024), respectively, to research articles related to ATD and CTD of cadmium. For the ECOTOX database (http://cfpub.epa.gov/ecotox, accessed on 20 August 2024), the retrieval strategy “Chemicals = (cadmium) and Effects = (all) and Endpoints = (LC_50_ and EC_50_ and NOEC and LOEC and MATC) and Species = (both animals and plants) and Test condition = (fresh water)” was employed to collect the ATD and CTD for cadmium to protect aquatic organisms. Additionally, both ATD and CTD without water hardness were excluded due to the potential effect of water hardness on the toxicity of cadmium to aquatic organisms.

According to the technical guidelines for deriving FWQC for the protection of aquatic organisms [25], toxicity data for the derivation of FWQC were required to cover species from three trophic levels, and the primary producers must be included among the freshwater aquatic species. Both ATD and CTD were expected to cover at least ten species from specified taxonomic groups, including one Cyprinidae fish, one non-Cyprinidae fish, one zooplankton, one benthic non-fish animal, as well as one phytoplankton or aquatic vascular plant [30].

### 2.2. Hardness Adjustment for TD

An analysis of covariance should be utilized to account for the relationship between water quality characteristics and toxicity of contaminants during the derivation of the FWQC for the protection of aquatic organisms [29,30,46]. ATD and CTD of cadmium to aquatic organisms were determined to meet the requirements for analysis of covariance according to technical guidelines for deriving water quality criteria for aquatic organisms [29]. A least-squares regression analysis was conducted on the logarithms of ATD and CTD in relation to the logarithms of water hardness for *H_A_* and *H_C_*, resulting in the pooled slope (*K_ATD_* and *K_CTD_*) as expressed in Equations (1) and (2). The water hardness adjustment of cadmium *ATD* and *CTD* for aquatic organisms was calculated in Equations (3) and (4) with K_ATD_ and K_CTD_, respectively.

(1) $$\mathrm{lg}(ATD)=K_{ATD}\mathrm{lg}(H_{A})+C_{A}$$

(2) $$\mathrm{lg}(CTD)=K_{CTD}\mathrm{lg}(H_{C})+C_{C}$$

(3) $$ATD_{H}=10^{k_{ATD}lg(ATD)-k_{ATD}lg(H_{A})}$$

(4) $$CTD_{H}=10^{k_{CTD}lg(CTD)-k_{CTD}lg(HC)}$$

where *H* was represented as the water hardness, with values of 50, 100, 150, 200, 250, 300, 350, and 450 mg/L. *H_A_* was the original water hardness value for ATD, and *H_C_* was the original water hardness value for CTD. The *ATD_H_* and *CTD_H_* were the TD adjusted to the corresponding water hardness, respectively. The *C_A_* and *C_C_* were the acute and chronic toxicity constants, respectively.

### 2.3. Statistical Analysis and the S-FWQC/L-FWQC Derivation by SSD Method

The SSD model was employed to calculate the S-FWQC and L-FWQC for the protection of aquatic organisms based on ATD and CTD of cadmium, respectively. The geometric mean of the available ATD and CTD for each species were designed as the species geomean acute toxicity data (SMAD) and species geomean chronic toxicity data (SMCD) as Equations (5) and (6), respectively.

(5) $$SMAD_{H,i}=\sqrt[{m}]{{\left(ATD_{H}\right)}_{i,1}\times {\left(ATD_{H}\right)}_{i,2}\times \ldots \times {\left(ATD_{H}\right)}_{i,m}}$$

(6) $$SMCD_{H,i}=\sqrt[{n}]{{\left(CTD_{H}\right)}_{i,1}\times {\left(CTD_{H}\right)}_{i,2}\times \ldots \times {\left(CTD_{H}\right)}_{i,n}}$$

where *m* and *n* were the total number of *ATD* and *CTD* for certain species.

Next, the *SMAD_H,i_/SMCD_H,i_* for all species were sorted in ascending order, with ranks assigned from the smallest (*R* = 1) to the largest (*R* = N). The cumulative probability (*F_R_*) for each species was then determined using Equation (7):

(7) $$F_{R}=\frac{\sum_{1}^{R}f}{\sum f+1}\times 100\%$$

where *f* was the number of species corresponding to the toxicity data rank *R*.

Four different models of the normal distribution model, log-normal distribution model, logistic distribution model, and log-logistic distribution model were employed to derive the S-FWQC/L-FWQC by plotting the logarithms of *SMAD_H.i_/SMCD_H.i_* as the independent variable and the cumulative probability of species as the dependent variable. Determined by the coefficient of determination *R*^2^, the most suitable model was applied to estimate the hazardous concentration of 5% (HC_5_) of freshwater species to protect the remaining 95% of species in the freshwater ecosystem. The S-FWQC and L-FWQC were calculated by dividing the acute and chronic HC_5_ values by an assessment factor (AF) ranging from 2 to 5 depending on the quantity and quality of the ATD and CTD.

**Figure 1:**
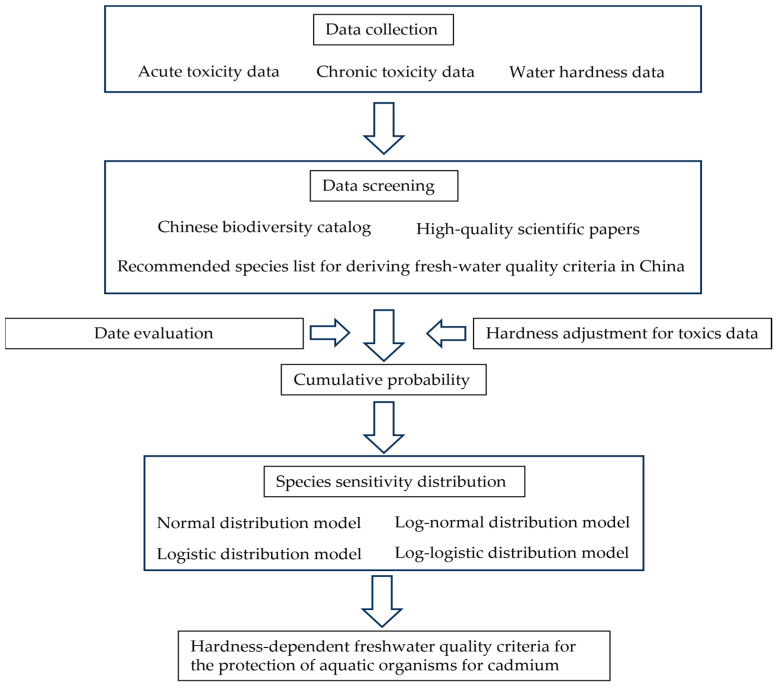
Flowchart of deriving hardness-dependent freshwater quality criteria for the protection of aquatic organisms for cadmium.

Typically, when the number of species exceeded 15 and covered a sufficient range of trophic levels for ATD and CTD, an AF of 2 was recommended according to the technical guideline for deriving water quality criteria for aquatic organisms [29]. Data analysis was conducted using EEC-SSD (Version 1.0, Ministry of Ecology and Environment, Beijing, China) and Origin 2022 for deriving the S-FWQC and L-FWQC (OriginLab, Northampton, MA, USA). The flowchart of FWQC for cadmium was shown in Figure 1.

## 3. Results and Discussion

### 3.1. The Database of ATD and CTD of Cadmium to Aquatic Organisms

**Figure 2:**
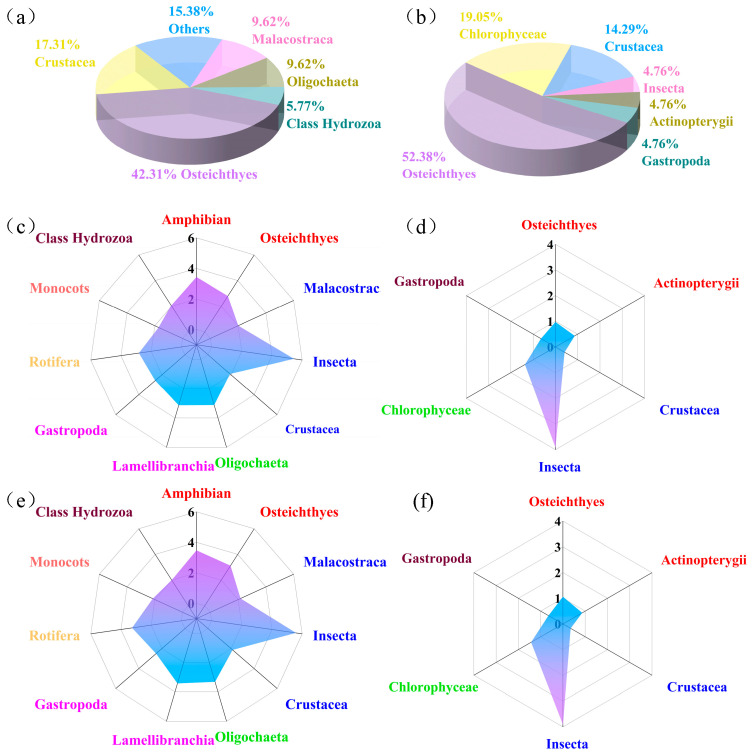
The proportion of freshwater aquatic organisms of different classes in acute toxicity data (**a**) and chronic toxicity data in China (**b**), the species geomean acute toxicity data (**c**), the species geomean chronic toxicity data (**d**), the species geomean acute toxicity data with water hardness of 100 mg/L as CaCO_3_ (**e**), and the species geomean chronic toxicity data with water hardness of 100 mg/L as CaCO_3_ (**f**) of cadmium to various classes groups of freshwater aquatic organisms (concentrations in μg/L) in China. The same-colored classes in (**c**–**f**) represent the same phylum.

The toxicity databases for cadmium were compiled from the literature and existing toxicity databases, involving 249 ATD for 52 species with the corresponding water hardness of aquatic organisms in China, as presented in Table 1. For acute toxicity, ATD were available for one freshwater plant, 30 invertebrate species (including nine planktonic and 21 benthic species), and 21 vertebrate species (including five cyprinid and 16 non-cyprinid teleost fishes) (Figure 2a). Regarding chronic toxicity, 62 CTD were identified for 21 species with the corresponding water hardness, which included one freshwater plant, five invertebrate species (three planktonic and two benthic species), and 12 vertebrate species (two cyprinid and 10 non-cyprinid teleost fishes) in China (Table 2 and Figure 2b). The data toxicity of cadmium to aquatic organisms adequately met the requirements for the derivation of ATD and CTD according to the technical guideline for deriving water quality criteria for freshwater organisms [29].

The percentages of Osteichthyes, Crustacea, Malacostraca, Oligochaeta, Hydrozoa, and other classes related to the ATD of cadmium to aquatic organisms were 42.31%, 17.31%, 9.62%, 9.62%, 5.77%, and 15.38%, respectively (Figure 2a). The SMAD for cadmium were ranked in ascending order as follows: Monocots < Crustacea < Malacostraca < Class Hydrozoa < Gastropoda < Osteichthyes < Rotifera < Oligochaeta < Lamellibranchia < Amphibian < Insecta (Figure 2c). Similarly, the percentages of Osteichthyes, Chlorophyceae, Crustacea, Insecta, Actinopterygii, and Gastropoda related to the CTD of cadmium to aquatic organisms were 52.38%, 19.05%, 14.29%, 4.76%, 4.76%, and 4.76%, respectively (Figure 2b). The SMCD for cadmium were ranked in ascending order as follows: Crustacea < Gastropoda < Actinopterygii < Osteichthyes < Chlorophyceae < Insecta (Figure 2d).

### 3.2. Derivation of the FWQC for Cadmium

#### 3.2.1. Correlations Between Water Hardness and Toxicity of Cadmium

**Figure 3:**
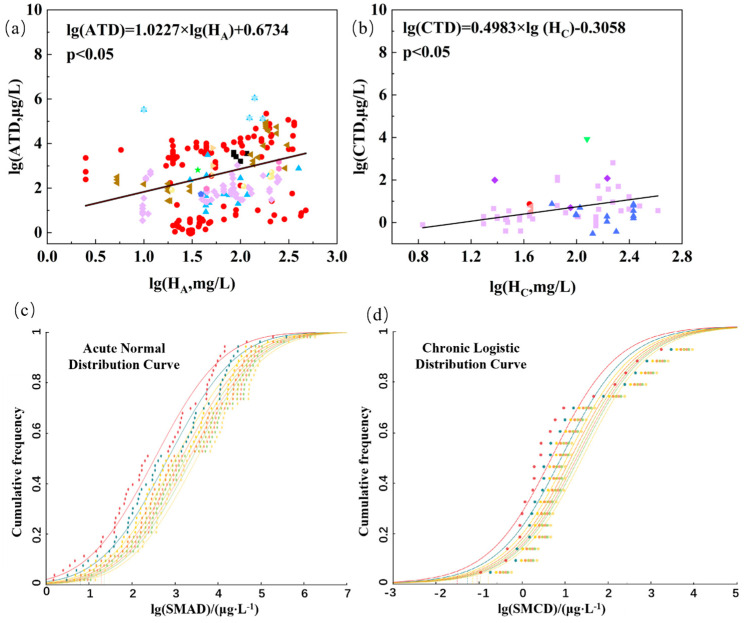
The linear relationship between the water hardness and (**a**) acute toxicity data (ATD) of 249 and (**b**) chronic toxicity data (CTD) of 62 of cadmium in China; different colors represent different classes. (**c**) the species sensitivity distribution (SSD) curves of cadmium ions of the logarithms of species geomean acute toxicity data [lg(SMAD)]; and (**d**) the logarithms of species geomean chronic toxicity data [lg(SMCD)] with different water hardness levels of 50, 100, 150, 200, 250, 300, 350, and 450 mg/L as CaCO_3_ from left to right.

A significant correlation was established between water hardness and the ATD of cadmium, with a slope (K_ATD_) of 1.0227 and a coefficient of determination (*R*^2^) of 0.1031 (*p* < 0.05) (Figure 3a). Additionally, a significant correlation was observed between water hardness and the CTD of cadmium, with a slope (K_CTD_) of 0.4983 and a coefficient of determination (*R*^2^) of 0.0448 (*p* < 0.05) (Figure 3b).

The TD of cadmium to freshwater aquatic organisms were influenced by the presence of calcium and magnesium ions because of the same valence and similar biological targets of aquatic organisms, including both ATD and CTD as mentioned above [39,44,47,48,49]. Consequently, cadmium exhibited greater toxicity in soft water than that in hard water for both short-term and long-term exposures to aquatic organisms. This result was also reported by the aquatic life ambient water quality criteria for cadmium published by the USEPA in 2016 [28]. Furthermore, the water hardness affected cadmium toxicity significantly in aquatic environments, which was consistent with the FWQC for the protection of aquatic organisms for cadmium released by the MEE in China in 2020 based on the characteristics of aquatic biota in China [49].

#### 3.2.2. Hardness-Dependent FWQC for Cadmium

According to the third national evaluation on surface water quality in China, the distribution of water hardness across the total surface water area was characterized by proportions of 42% for a water hardness below 150 mg/L, 34% for levels between 150 and 300 mg/L, 11% for levels between 300 and 450 mg/L, and 13% for levels above 450 mg/L, respectively [30,50]. Utilizing the principle of equal data distribution, ATD and CTD were adjusted to eight distinct water hardness using the correction equation (Equation (2)) along with the K_ATD_ and K_CTD_ coefficients, respectively. The range of ATD for cadmium was found to be from 0.9 µg/L to 1,106,000 µg/L with water hardness levels varying from 2.5 mg/L to 475 mg/L as CaCO_3_ (Table 1). The range of CTD for cadmium was identified as from 0.3 µg/L to 8,249 µg/L with water hardness values from 6.8 mg/L to 340 mg/L as CaCO_3_ (Table 2). Following the water hardness adjustment, the SMAD_100,i_ ranged from 3.05 μg/L to 389,447.18 μg/L (Table 1), and the SMCD_100,i_ varied from 0.79 μg/L to 7532.61 μg/L with a water hardness reference of 100 mg/L as CaCO_3_ (Table 2). The percentages of species and the SMAD_100,i_/SMAD_100,i_ of cadmium were similar to those before water hardness correction (Figure 2e,f).

Following the adjustment of the SMAD_H,i_ for water hardness, the three species identified as most sensitive to cadmium among the 52 species were non-cyprinid teleost fish: *Morone saxatilis*, *Oncorhynchus mykiss*, and *Salvelinus confluentus*. Conversely, the three species exhibiting the most tolerance to cadmium were *Branchiura sowerbyi*, *Pseudorasbora parva*, and *Chironomus riparius* based on short-term exposure (Table 1). After the adjustment of SMCD_H,i_, the three species most sensitive to cadmium among the 21 species were *Daphnia magna*, *Oncorhynchus mykiss*, and *Ceriodaphnia dubia* based on long-term exposure. The three species demonstrating the most tolerance to cadmium were *Chlamydomonas Reinhardtii*, *Cyprinus carpio*, and *Pachydiplax longipennis* as a comparison of the SMCD in China (Table 2). Therefore, *Daphnia magna* might be used as an indicator species for cadmium pollution in the aquatic environment in China.

The most sensitive species identified were *Salvelinus confluentus*, *Cottus bairdii*, and *Salmo trutta*, while the most tolerant species were *Chironomus plumosus* and *Cyprinus carpio* for short-term exposure according to the aquatic life ambient water quality criteria for cadmium published by the USEPA in 2016 (Table 3) [19,28]. For long-term exposure, the most sensitive species included *Hyalella azteca*, *Ceriodaphnia dubia*, and *Cottus bairdii*, whereas the most tolerant species were *Aeolosoma headleyi* and *Oreochromis aureus* in the USA based on the aquatic life ambient water quality criteria for cadmium (Table 3) [19,28]. Therefore, the different sensitive and tolerant species for cadmium in the freshwater ecosystem indicated the difference in aquatic biota between China and the USA.

By using the SMAD_H,i_ and cumulative probability as independent and dependent variables, the *R*^2^ values for the normal, log-normal, logistic, and log-logistic distribution models were calculated as 0.9793, 0.9786, 0.9785, and 0.9747, respectively, for the SMAD_100,i_ of aquatic organisms in China (Table S2). Similarly, the SSD curves were applied to fit SMCD_100,i_, resulting in *R*^2^ values of 0.9644 and 0.9686 for the normal and logistic distribution models, respectively (Table S2). Therefore, the normal distribution model and logistic distribution model were the best models fit for the SMAD_H,i_ and SMCD_H,i_ of cadmium, respectively, to derive the FWQC. The SSD curves and HC_5_ showed a rightward shift as water hardness increased for both the SMAD_H,i_ and SMCD_H,i_, suggesting that both the ATD and CTD of cadmium decreased with increasing water hardness (Figure 3c,d).

The acute HC_5_ value of cadmium ranged from 3.12 to 29.52 µg/L, while the chronic HC_5_ value ranged from 0.25 to 0.74 µg/L with water hardness levels varying from 50 to 450 mg/L as CaCO_3_ (Table 4 and Table S3). An assessment factor of 2 was established for the toxicity data covering 15 species for both ATD and CTD, which was in accordance with the technical guidelines for deriving water quality criteria in China. This assessment factor aligns with the FWQC for the protection of aquatic organisms for cadmium published by the MEE in China in 2020 [30].

Consequently, the S-FWQC for cadmium were calculated in a range of 1.56 to 14.76 µg/L at a water hardness of 50–450 mg/L as CaCO_3_ (Table 4). The L-FWQC for cadmium ranged from 0.12 to 0.37 µg/L at a water hardness of 50–450 mg/L as CaCO_3_ (Table 4). Notably, it was observed that the ratio of S-FWQC to L-FWQC at the lowest water hardness level (50 mg/L) was nearly 9/3 times greater than that at the highest water hardness level (450 mg/L) in China. This highlights the importance of considering water hardness during deriving both S-FWQC and L-FWQC to protect aquatic organisms from cadmium exposure. The S-FWQC and L-FWQC of cadmium can also be expressed with the equation $10^{\left(1.0227\times lg(H)-1.5444\right)}$ and $10^{\left(0.4983\times lg(H)-1.7549\right)}$, respectively, in China. In detail, the derived S-FWQC (3.17 µg/L) in this research was 24.5% less than that (4.2 µg/L) published by MEE in China, while L-FWQC (0.17 µg/L) in this research was 26.1% less than that (0.23 µg/L) published by MEE in China at the water hardness of 100 mg/L as CaCO_3_ [30]. Therefore, it is necessary to eliminate invasive species and internationally common species without distribution in China during the derivation of FWQC for the protection of aquatic organisms.

### 3.3. The Comparison with Other FWQC and WQSs for Cadmium

**Figure 4:**
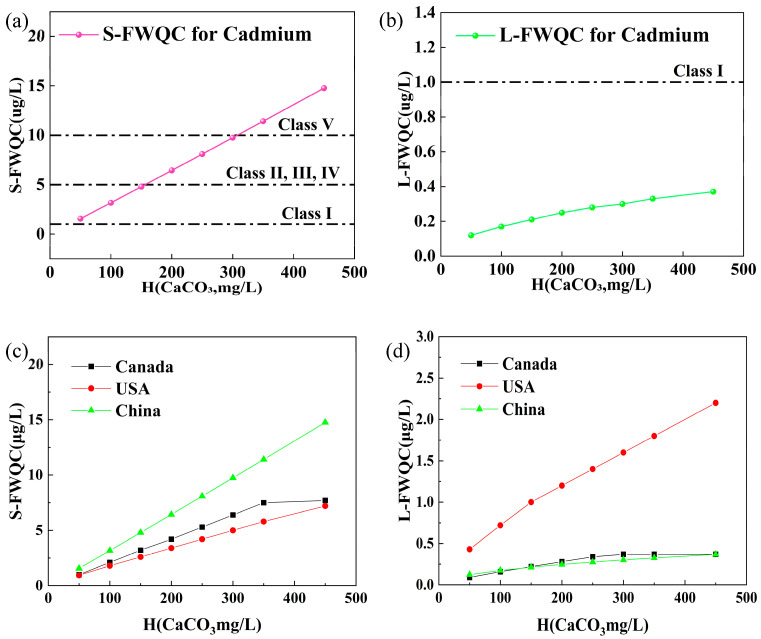
Comparison of the limits of GB3838-2002 and the short-term freshwater quality criteria (S-FWQC) (**a**) and long-term freshwater quality criteria (L-FWQC) (**b**) of cadmium for the protection of aquatic organisms. The change in S-FWQC (**c**) and L-FWQC (**d**) in China, Canada, and USA with water hardness.

The FWQC for the protection of aquatic organisms for cadmium were investigated and published by several countries, including the USA, Canada, Australia, New Zealand, EU, etc. The criterion maximum concentration (CMC) and criterion continuous concentration (CCC) for cadmium were expressed as $e^{\left(0.9789\times \ln(H)-3.866\right)}$ and $e^{\left(0.7977\times \ln(H)-3.909\right)}$, respectively, with water hardness serving as the independent variable for the protection of aquatic organisms issued by the USEPA [28]. In Canada, the S-FWQC and L-FWQC were given by $10^{\left(1.016\times log(H)\right)}$ and $10^{\left(0.83\times log(H)-2.46\right)}$ for the water quality guidelines of cadmium with water hardness as the independent variable [21]. With a water hardness of 100 mg/L as CaCO_3_, S-FWQC (3.17 µg/L) in the present research was 76.1% greater than the CMC (1.8 µg/L) in the USA and was 26.8% greater than that in Canada (2.5 µg/L) (Figure 4c). The L-FWQC (0.17 µg/L) in the present research was 76.4% less than the CMC (0.72 µg/L) in the USA and was similar to that in Canada (0.18 µg/L), with a water hardness of 100 mg/L as CaCO_3_ (Figure 4d). According to the freshwater quality guidelines in Australia and New Zealand, the trigger value for cadmium was set at 0.2 µg/L, with a chosen protection level of 95% (30 mg/L), applicable to moderately hard water at 90 mg/L (as CaCO_3_) [22], which was greater than the L-FWQC of 0.17 µg/L determined in this research at a water hardness of 100 mg/L. Environmental quality standards (EQSs) were established to protect aquatic organisms by the EU, including the annual average concentration (AA-EQS) and the maximum allowable concentration (MAC-EQS) [16]. Both the AA-EQS and MAC-EQS increased with the increase in water hardness from 40 mg/L to 200 mg/L, which was similar to the variation in the CMC and CCC in the USA and the S-FWQC and L-FWQC in China in this study. The differences in water hardness range and classification are primarily due to the distribution range of water hardness in China and the EU. In detail, according to the third national surface water quality assessment in China, a surface water hardness <150 mg/L, 150 mg/L~<300 mg/L, 300 mg/L~≤450 mg/L, and >450 mg/L accounted for 42%, 34%, 11%, and 13% in China, respectively. The S-FWQC (3.17 µg/L) in the present research was 2.5 times greater than the MAC-EQS (0.9 µg/L) in the EU, and the L-FWQC (0.17 µg/L) was approximately comparable to the AA-EQS (0.15 µg/L) with a water hardness of 100 mg/L as CaCO_3_. The variations in water quality standards/criteria among different countries and international organizations might be attributed to differences in aquatic biota, freshwater conditions, and toxicity data. Therefore, it is essential to derive the FWQC from the toxicity data of native species to minimize the potential influence of water characteristics and specific taxonomic groups of species in different countries and regions.

At present, the permissible limits for cadmium were set at 1 µg/L for Class I, 5 µg/L for Classes II, III, and IV, and 10 µg/L for Class VI for the GB3838-2002 [25]. Under short-term exposure at a certain water hardness of 100 mg/L of CaCO_3_, the S-FWQC of 3.17 µg/L was 3.17 times greater than Class I (1 µg/L) of GB3838-2002, 36.6% less than Classes II-IV (5 µg/L) of GB3838-2002, and 68.3% times less than Class V (10 µg/L) of GB3838-2002. Under Class II-IV of GB3838-2002, *Morone saxatilis* and *Oncorhynchus mykiss* might experience adverse effects by cadmium to aquatic organisms with short-term exposure at an arbitrary water hardness of 100 mg/L of CaCO_3_. Under Class V of GB3838-2002, *Morone saxatilis*, *Oncorhynchus mykiss*, *Salvelinus confluentus*, *Salmo trutta*, and *Oncorhynchus tshawytscha* might be affected by cadmium to aquatic organisms with short-term exposure at an arbitrary water hardness of 100 mg/L of CaCO_3_ at least. In long-term exposure at a certain water hardness of 100 mg/L of CaCO_3_, the L-FWQC of 0.17 µg/L was 83% less than Class I (1 µg/L) of GB3838-2002, 96.6% less than Classes II-IV (5 µg/L) of GB3838-2002, and 98.3% less than Class V (10 µg/L) of GB3838-2002. Under Class I of GB3838-2002, *Daphnia magna* may be harmed by cadmium to aquatic organisms with long-term exposure at an arbitrary water hardness of 100 mg/L of CaCO_3_. Under Class II-IV of GB3838-2002, *Daphnia magna*, *Oncorhynchus mykiss*, *Ceriodaphnia dubia*, *Oncorhynchus kisutch*, *Oryzias latipes*, *Oncorhynchus tshawytscha*, and *Salvelinus fontinalis* were likely to be adversely affected by cadmium to aquatic organisms with long-term exposure at an arbitrary water hardness of 100 mg/L of CaCO_3_ at least. Under Class V of GB3838-2002, 13 species might suffer harm by cadmium to aquatic organisms with long-term exposure at an arbitrary water hardness of 100 mg/L of CaCO_3_, including *Aplexa hypnorum*, *Daphnia pulex*, *Salmo salar*, etc., shown as rank1-13 in Table 2. The limit of cadmium of GB3838-2002 and the environmental risks of cadmium require careful consideration in the freshwater ecosystems in China based on the L-FWQC.

Water hardness was considered a key factor in deriving the FWQC to support the scientific management and assessment of water quality. The S-FWQC at a hardness of 500 mg/L (14.76 µg/L) was about 10 times greater than at a hardness of 50 mg/L (1.56 µg/L), while the L-FWQC at the hardness of 500 mg/L (0.37 µg/L) was about 3 times greater than at a hardness of 50 mg/L (0.12 µg/L) in China. Additionally, invasive species and internationally common species without distribution in China were excluded from the FWQC for cadmium to ensure its greater applicability to unique ecosystems. The toxicity database for cadmium covered 249 ATD from 52 species and 62 CTD from 21 species to derive the FWQC in China. Abundant aquatic species exist in the surface water in China (more than 20,000 species), and less than 100 species were applied to derive the FWQC to protect the aquatic organisms in China. Therefore, there was an urgent need to supplement the TD of cadmium for native species in China, particularly the CTD, to improve the accuracy and applicability of the FWQC. Water hardness was considered the primary factor in this study because the cadmium toxicity varied with the changes in water hardness for both the ATD and CTD in China. However, other water quality parameters, such as pH, dissolved oxygen, temperature, organic matter, and other metal ions, might affect the toxicity of cadmium to protect aquatic organisms. Due to the lack of TD for cadmium under controlled experimental conditions, such as pH, dissolved oxygen, temperature, organic matter, and other metal ions, more research studies are needed to investigate the effects of these additional factors on aquatic organisms. By utilizing the hardness-based FWQC, a nationwide ecological risk assessment for cadmium can be conducted to protect aquatic organisms in surface water in China. The re-evaluation of cadmium remediation technologies, such as adsorption technology, precipitation technology, and microbial remediation, should also be conducted for potential application in the treatment of environmental pollution in surface waters of rivers, lakes, and reservoirs based on the FWQC in China.

## 4. Conclusions

The ATD of 249 from 52 species and the CTD of 62 from 21 species were applied to establish the FWQC for the protection of aquatic organisms for cadmium in China, excluding invasive species and internationally common species without distribution in China. Significant correlations between the water hardness and toxicity databases of cadmium were obtained with a K_ATD_ of 1.0227 (n = 52, *p* < 0.05) for the ATD and a K_CTD_ of 0.4983 (n = 21, *p* < 0.05) for the CTD, respectively, in China. Among the studied species, *Morone saxatilis* exhibited the most sensitivity for cadmium during short-term exposure, while *Daphnia magna* demonstrated the most sensitivity for cadmium during long-term exposure, which made it a potential indicator of cadmium contamination in the freshwater ecosystems under long-term exposure. The S-FWQC and L-FWQC can be expressed using the equation $10^{\left(1.0227\times lg(H)-1.5444\right)}$ and $10^{\left(0.4983\times lg(H)-1.7549\right)}$, respectively, with water hardness as CaCO_3_ as an independent variable. The best fitting models of the normal distribution model and logistic distribution model were selected to derive the S-FWQC and L-FWQC, respectively, and were calculated to be 1.56–14.76 µg/L and 0.12–0.37 µg/L at a water hardness of 50–450 mg/L as CaCO_3_. The possible environmental risk and cadmium remediation technologies should be established and given more concern regarding the FWQC of cadmium in freshwater to protect the aquatic organisms in China, especially with the L-FWQC.

## Figures and Tables

**Table 1:** Ranked freshwater aquatic organisms of the species geomean acute toxicity data with water hardness of 100 mg/L as CaCO_3_ (SMAD_100.i_) and acute toxicity data (ATD) of cadmium to freshwater aquatic organisms in China.

Rank	Species	N	Hardness (mg/L)	ATD (μg/L)	SMAD_100,i_
1	*Morone saxatilis*	3	40–475	4–10	3.05
2	*Oncorhynchus mykiss*	7	20–427	2.07–7.56	3.21
3	*Salvelinus confluentus*	13	29.3–31.7	0.9–6.6	5.14
4	*Salmo trutta*	13	29.2–151	1.23–15.1	7.34
5	*Oncorhynchus tshawytscha*	6	21–343	1.1–57	9.61
6	*Oncorhynchus kisutch*	5	22–90	2–17.5	15.73
7	*Gammarus pulex*	2	94.6–117.4	20–50	29.97
8	*Duphnia magna*	6	30–250	30–244	38.24
9	*Hydra viridissima*	2	19.5–210	3–210	39.62
10	*Cherax quadricarinatus*	3	43.79	8.48–44.8	43.18
11	*Ceriodaphnia dubia*	8	40–172	31.47–361.1	79.28
12	*Gammarus pseudolimnaeus*	5	43.5–76.8	22–68.3	81.23
13	*Ceriodaphnia rericulata*	6	45–240	66–184	82.20
14	*Simocephalus vetulus*	2	45–67	24–89.3	85.47
15	*Daphnia pulex*	9	40–240	44.96–99	103.16
16	*Simocephalus serrulatus*	9	9.7–67	3.5–123	105.69
17	*Moina macrocopa*	5	82.00	71.25–412	133.82
18	*lemra minor*	1	39.00	650.00	141.45
19	*Hydra oligactis*	1	210.00	320.00	149.84
20	*Hydra vulgaris*	5	19.5–210	82.5–520	167.75
21	*Aplexa hypnorum*	2	44.4–44.8	93.00	212.38
22	*Neocaridina denticulate*	4	30–400	230–2592	299.04
23	*Oryzias latipes*	2	50–100	130–350	304.04
24	*Diaphanosoma brachyurum*	2	67.1–93	69.8–1060	346.18
25	*Nais elinguis*	5	17.89–18.72	27–158	347.60
26	*Lumbriculus variegatus*	5	10–290	74–780	408.34
27	*Lymnaea stagnalis*	3	250.00	752–1585	477.07
28	*Chydorus sphaericus*	6	10.5–83.6	149–560	1419.18
29	*Limnodrilus hoffmeisteri*	4	5.3–152	170–2400	1542.59
30	*Brachionus calyciflorus*	1	36.20	650.00	1837.48
31	*Anguilla rostrata*	3	55.00	820–1500	2038.44
32	*Procambarus acutus*	11	85.5–262.5	1390–7160	2397.29
33	*Hyriopsis cumingii*	3	51.43	388–6346	2669.07
34	*Prychocheilus oregonensis*	4	25–347	1092–5555	2715.64
35	*Bufo gargarizans*	1	90.00	2592.00	2886.90
36	*Xenopus laevis*	4	85–116	1600–4000	3064.99
37	*Poecilia reticulata*	9	18.72–209.2	170–16,000	4691.95
38	*Tubifex tubifex*	6	5.3–305	320–56,000	6150.98
39	*Gasterosteus aculeatus*	2	107.15–115	6500–23,000	10,988.70
40	*Tanichthys albonubes*	2	39.16–44.5	4610–4447	11,063.56
41	*Ctenopharyngod on idella*	5	42.72–210.1	3490–24,500	11,348.44
42	*Oreochromis mossambica*	2	17–28.4	1000–6000	11,538.02
43	*Ictalurus punctatus*	5	44.4–67	4610–10,200	12,849.68
44	*Carassius auratus*	4	20–144	2130–46,800	14,828.01
45	*Lepomis macrochirus*	16	20–350	1700–48,200	15,968.42
46	*Cyprinus carpio*	8	100–312.5	6500–220,770	18,273.53
47	*Lepomis cyanellus*	7	20–360	2840–88,600	19,967.71
48	*Cirrhinus mrigala*	2	19.5–72	5300–13,700	23,253.70
49	*Aristichthys nobilis*	3	2.50	245–2250	29,040.73
50	*Branchiura sowerbyi*	10	5.3–195	240–88,780	29,461.18
51	*Pseudorasbora parva*	1	5.80	5170.00	95,089.54
52	*Chironomus riparius*	4	10–170	128,840–1,106,000	389,447.18

N is the number of ATD collected in the literature and toxicity databases.

**Table 2:** Ranked freshwater aquatic organisms of the species geomean chronic toxicity data with water hardness of 100 mg/L (SMCD_100,i_) as CaCO_3_ and chronic toxicity data (CTD) of cadmium to freshwater aquatic organisms in China.

Rank	Species	N	Hardness (mg/L)	CTD (μg/L)	SMCD_100,i_
1	*Daphnia magna*	5	99–200	0.3–2.39	0.79
2	*Oncorhynchus mykiss*	17	6.8–413.8	0.4–4.31	1.94
3	*Ceriodaphnia dubia*	7	100–270	1.602–6.257	2.04
4	*Oncorhynchus kisutch*	1	44.00	2.10	3.16
5	*Oryzias latipes*	2	340.00	50.00	3.17
6	*Oncorhynchus tshawytscha*	2	25.00	1.57–1.88	3.43
7	*Salvelinus fontinalis*	4	37–188	2.045–9.165	3.82
8	*Salmo trutta*	7	30.6–250	0.4–16.49	5.01
9	*Scenedesmus acutus*	1	90.00	5.00	5.27
10	*Chlorella vulgaris*	1	90.00	5.00	5.27
11	*Aplexa hypnorum*	2	45.30	3.46–5.801	6.65
12	*Daphnia pulex*	2	65–106	5–7.49	6.71
13	*Salmo salar*	1	23.50	4.53	9.32
14	*Esox lucius*	1	44.00	7.36	11.08
15	*Lepomis macrochirus*	3	147–207	4.167–49.8	15.00
16	*Oreochromisaurea*	1	145.00	52.00	43.21
17	*Pseudokirchneri ella subcapitata*	1	171.00	120.00	91.85
18	*Cirrhinus mrigala*	2	71.50	98–132	134.43
19	*Chlamydomonas Reinhardii*	1	24.00	99.00	201.59
20	*Cyprinus carpio*	1	188.50	650.00	473.94
21	*Pachydiplax longipennie*	1	120.00	8249.00	7532.61

N is the number of CTD collected in the literature and toxicity databases.

**Table 3:** Three sensitive species of cadmium in different countries (China, Canada, USA) in short-term exposure and long-term exposure.

Nation	Short-Term Exposure	Long-Term Exposure
China	*Morone Saxatilis*;	*Daphnia magna*;
	*Oncorhynchus mykiss*	*Oncorhynchus mykiss*
	*Salvelinus confluentus*;	*Ceriodaphnia dubia*;
Canada	*Oncorhynchus mykiss*;	*Daphnia magna*;
	*Hyalella Azteca*;	*Ceriodaphnia reticulata*;
	*Daphnia magna*	*Hyalella Azteca*;
USA	*Salvelinus confluentus*;	*Hyalella Azteca*;
	*Cottus bairdii*;	*Ceriodaphnia dubia*;
	*Salmo trutta*	*Cottus bairdii*

**Table 4:** Hazardous concentration of 5% (HC_5_) and short-term freshwater quality criteria (S-FWQC)/long-term freshwater quality criteria (L-FWQC) of cadmium for different water hardness deriving with the species sensitivity distribution model.

	H (CaCO_3_, mg/L)	HC_5_ (μg/L)	FWQC (μg/L)
S-FWQC	50	3.12	1.56
	100	6.34	3.17
	150	9.60	4.80
	200	12.88	6.44
	250	16.18	8.09
	300	19.50	9.75
	350	22.83	11.41
	450	29.52	14.76
L-FWQC	50	0.25	0.12
	100	0.35	0.17
	150	0.43	0.21
	200	0.49	0.25
	250	0.55	0.28
	300	0.60	0.30
	350	0.65	0.33
	450	0.74	0.37

## Data Availability

The data will be made available on request.

## Supplementary Materials

The following supporting information can be downloaded at https://www.mdpi.com/article/10.3390/toxics12120892/s1, Table S1. List of Abbreviations; Table S2. Fitting results of short-term freshwater quality criteria and long-term freshwater quality criteria species sensitivity distribution model with water hardness of 100 mg/L as CaCO_3_; Table S3. Hazard concentration of short-term species and long-term species.
