# Supplementary material for: Hardness-Dependent Freshwater Quality Criteria for the Protection of Aquatic Organisms for Cadmium in China
Source: Toxics. 2024 Dec 8;12(12):892. doi: 10.3390/toxics12120892 (PMC11728563; doi:10.3390/toxics12120892)
Supplement: Supplementary file 1 [file toxics-12-00892-s001.zip › toxics-3319404-supplementary.pdf]

Supporting Information

Hardness-Dependent Freshwater Quality Criteria for the Protection of Aquatic Organisms for Cadmium in China

Zeya Zhang[1], Rui Huang[1], Zhongjie Shen[1,2], Yili Fan[1,3], Chenglian Feng[1], Yingchen Bai[1,*]

[1] State Key Laboratory of Environmental Criteria and Risk Assessment, Chinese Research Academy of Environmental Sciences, Beijing, 100012, China

[2] School of Environmental Science and Engineering, Changzhou University, Changzhou 213164, China

[3] College of Water Science, Beijing Normal University, Beijing 100875, China

[*]Corresponding author:

Yingchen Bai Ph.D

State Key Laboratory of Environmental Criteria and Risk Assessment, Chinese Research Academy of Environmental Sciences, Beijing, 100012, China

Tel: +86-10-84913674

Fax: +86-10-84913674

Email: baiyc@craes.org.cn

Table S1. List of abbreviations.

Number	Abbreviation	Full Term
1	FWQC	Freshwater quality criteria
2	K_{ATD}	Acute toxicity coefficient
3	K_{CTD}	Chronic toxicity coefficient
4	S-FWQC	Short-term freshwater quality criteria
5	L-FWQC	Long-term freshwater quality criteria
6	WQS	Water quality standards
7	SSD	Species sensitivity distribution
8	TD	Toxicity data
9	ATD	Acute toxicity data
10	CTD	Chronic toxicity data
11	LC_{50}	Median lethal concentration
12	EC_{50}	Median effect concentration
13	NOEC	No observed effect concentration
14	LOEC	Lowest observed effect concentration
15	MATC	Maximum acceptable toxicant concentration
16	EC_{10}	10% effect concentration
17	EC_{20}	20% effect concentration
18	H_A	Original water hardness value for ATD
19	H_C	Original water hardness value for CTD
20	ATD_H	ATD adjusted to the corresponding water hardness
21	CTD_H	CTD adjusted to the corresponding water hardness
22	C_A	Acute toxicity constants
23	C_C	Chronic toxicity constants
24	SMAD	Species geomean acute toxicity data
25	SMCD	Species geomean chronic toxicity data
26	F_R	Cumulative probability
27	HC_5	Hazardous concentration of 5%
28	AF	Assessment factor
29	CMC	Criterion maximum concentration
30	CCC	Criterion continuous concentration
31	EQS	Environmental quality standards
32	AA-EQS	Annual average concentration
33	MAC-EQS	Maximum allowable concentration
34	EU	European Union

Table S2. Fitting results of short-term freshwater quality criteria and long-term freshwater quality criteria species sensitivity distribution model with water hardness of 100 mg/L as $CaCO_3$.

Term	Fitting Model	R^2	RMSE	SSE
Short-term	Normal distribution model	0.9793	0.0443	0.1221
	Log-Normal distribution model	0.9786	0.0638	0.1266
	Logistic distribution model	0.9785	0.0487	0.1269
	Log-Logistic distribution model	0.9747	0.0474	0.1498
Long-term	Norma distribution model	0.9644	0.0955	0.0693
	Logistic distribution model	0.9686	0.0785	0.4707

Table S3. Hazard concentration of short-term species and long-term species.

Term	H (以 CaCO3 计,mg/L)	HC_X(μg/L)						
		HC_5	HC_{10}	HC_{25}	HC_{50}	HC_{75}	HC_{90}	HC_{95}
Short-term	50	3.12	1.56	11.22	8.80	60.39	49.80	391.60
	100	6.34	3.17	23.17	17.88	124.70	101.16	808.40
	150	9.60	4.80	35.41	27.07	190.50	153.14	1235.00
	200	12.88	6.44	47.84	36.33	257.30	205.54	1669.00
	250	16.18	8.09	60.41	45.65	325.00	258.23	2107.00
	300	19.50	9.75	73.10	55.00	393.20	311.17	2550.00
	350	22.83	11.41	85.88	64.40	462.00	364.33	2996.00
	450	29.52	14.76	111.70	83.27	600.90	471.09	3897.00
Long-term	50	0.25	0.12	0.69	0.58	2.30	2.05	7.63
	100	0.35	0.17	1.04	0.82	3.45	2.90	11.43
	150	0.43	0.21	1.32	1.01	4.37	3.55	14.48
	200	0.49	0.25	1.56	1.16	5.17	4.10	17.13
	250	0.55	0.28	1.78	1.30	5.89	4.58	19.51
	300	0.60	0.30	1.98	1.42	6.55	5.01	21.70
	350	0.65	0.33	2.16	1.54	7.16	5.41	23.74
	450	0.74	0.37	2.50	1.74	8.29	6.14	27.48
